# A Slashing Document

**Published:** 1920-04-10

**Authors:** 


THE PUBLIC WELL-BEING—(*continued*).

### A SLASHING DOCUMENT.

### American View of English Decadence.

We have received the report of an address delivered at a meeting of the National Civic Federation in America, at the end of last January, by Mr. Frederick L. Hoffman, LL.D., of the Prudential Insurance Company of America, on the methods and results of National Health Insurance in the United Kingdom. It is a slashing document of seventy-two printed pages, in which the whole thing is condemned root and branch on every conceivable ground and, according to the author, by every conceivable authority, except political opportunists. He says that Mr. Lloyd George introduced it without any popular desire for such a measure, that he went to the German Government for his information, and that the German Government, having already found itself penalised in the field of foreign competition by the financial burdens of national insurance, was naturally only too glad, in the course of its usual commercial schemes, to persuade any of its rivals to burden itself in the same way. National Health Insurance, we are told, is bureaucratic and autocratic; it deprives the individual of liberty, it wastes money, it deteriorates the status of the medical profession, it satisfies nobody, has no beneficial effects whatever, and is generally a sign of the decadence of English tradition for liberty.

In the midst of this gloomy consideration of an English measure it is refreshing to find one or two paragraphs which assert that English sanitary progress has, by every authority on the subject, been considered superior to the corresponding progress attained in any other section of the civilised world, and that the public health administration of England is a model for the civilised world. The lowering of the English death-rate during the last thirty years challenges favourable comparison, and this has been achieved without social insurance or compulsory health insurance of any kind, but rather in response to a realised understanding of the problems fairly within reach of successful solution. National Health Insurance, however, says the author, has fostered a tendency to lower public interest in public health as the direct result of the encouraging of malingering and the exaggeration of slight ailments, imposing an enormous burden upon the medical profession, as well as upon the public Treasury. He quotes every available authority he can lay hands on to support his view, but so far, at any rate, as it concerns the lowering of public interest in public health there is a mass of evidence to show that the direct contrary is the case. Never has public interest been so marked as it is now. It is not yet what it might be, but it is far in advance of what it has ever been.

### Extravagant Suggestion.

The pamphlet, as a matter of fact, seems largely designed as a propaganda effort against those who are anxious to adopt some national health insurance methods in the United States, and it is also marked by certain political nervousness regarding the trend of such State legislation. Nobody ever supposed that the National Health Insurance Act was all that it might have been, or that its provisions were adequate. But to suggest that the system reveals "most sinister dangers to democracy and liberty, has led to social stratification, and is unquestionably one of the principal causes of industrial unrest," is putting the case ridiculously high. The average politician and social reformer, if he were told that the principal causes of industrial unrest in England or Europe or anywhere else were to be found in a system of National Health Insurance, would undoubtedly receive it with a look of bland astonishment, while here this pamphleteer tells us that it is "a most subtle and deceptive method of undermining the efficacy of democratic institutions." It is rather difficult to discover why the scheme has ever been allowed to exist at all, for the pamphlet seems to embrace absolutely everybody in England in one deadly phalanx of hostility. It reads almost like the political controversies of those far-off days when the Act was being debated in Parliament.

This cheerless American prophet concludes that such insurance, viewed from the social, medical, or economic standpoint, must needs undermine the foundations of government and lead ultimately to the "disintegration and bankruptcy of the State." Happily, we who live under it do not entertain such despondency. It is true the author gives some alternative plan. He suggests, for instance, that the medical needs of the general population can be more effectively provided for through a system of public medicine than through State Insurance. Under a rational system of public medicine it would be left to the option of the individual to take advantage of it on a voluntary basis in precisely the same manner as use is now being made of public libraries, public baths, public playgrounds, parks, education, etc. Those who do not wish to make use of public facilities pay for private gardens, baths, schools, libraries, etc. To avoid undemocratic and dangerous class distinctions, a system of public medicine must be universal, with an absolutely free choice of State possessions reasonably available to the public needs.

### Compulsion and Liberty.

We like best the statement that "a thorough understanding of the methods and results of National Health Insurance in the United Kingdom can only be arrived at on the basis of a full and impartial inquiry." But in general we may be forgiven for becoming a little tired of so many paternal lectures from the other side of the ocean upon European institutions and ideals, and to be told continually, as this pamphlet tells us, that every European Government is encouraging further waste of time, money, and effort to serve only temporary political ends. At the same time, there is much in this clever exposition which is suggestive, a good deal that is true, and a lot that is exaggerated. But it is entirely erroneous to suggest or suppose that individual English liberty has deteriorated, and it is still true that no country in the world enjoys the same personal freedom as we do. Neither is it correct to assume that industrial unrest is any more marked here than in America or elsewhere; the evidence in most cases points in the contrary direction. We hold no brief for the Insurance Act. Everyone knows its defects, but they must be studied soberly, stated soberly, and handled soberly if any useful result is to follow. Rhetoric is of very little value, and when it is mixed with political polemics it is of still smaller account. And so far as compulsory health measures are concerned it is absurd to imagine that any well-ordered State can afford to dispense with them, or that in enforcing them, any more than in enforcing laws against burglary, it is robbing people of liberty. It is exactly the opposite; it gives them greater liberty by removing from them the chains of the ignorant and careless.

